# Estimation of national and subnational all-cause mortality indicators in Nepal, 2017

**DOI:** 10.1186/s12889-022-14638-z

**Published:** 2022-12-03

**Authors:** Surender Prasad Pandey, Tim Adair

**Affiliations:** 1 The Nossal Institute for Global Health, Melbourne School of Population and Global Health, University of Melbourne, Carlton, VIC 3053 Australia; 2 Ministry of Federal Affairs and General Administration, Kathmandu, Nepal

**Keywords:** Nepal, Civil Registration, Vital Statistics, Completeness of registration, Mortality, Subnational, Epidemiological transaction, Adult mortality, Life expectancy, Monetary poverty

## Abstract

**Background:**

Despite the civil registration and vital statistics (CRVS) system in Nepal operating for several decades, it has not been used to produce routine mortality statistics. Instead, mortality statistics rely on irregular surveys and censuses that primarily focus on child mortality. To fill this knowledge gap, this study estimates levels and subnational differentials in mortality across all ages in Nepal, primarily using CRVS data adjusted for incompleteness.

**Methods:**

We analyzed death registration data (offline or paper-based) and CRVS survey reported death data, estimating the true crude death rate (CDR) and number of deaths by sex and year for each province and ecological belt. The estimated true number of deaths for 2017 was used with an extension of the empirical completeness method to estimate the adult mortality (*45q15*) and life expectancy at birth by sex and subnational level. Plausibility of subnational mortality estimates was assessed against poverty head count rates.

**Results:**

Adult mortality in Nepal for 2017 is estimated to be 159 per 1000 for males and 116 for females, while life expectancy was estimated as 69.7 years for males and 73.9 years for females. Subnationally, male adult mortality ranges from 129 per 1000 in Madhesh to 224 in Karnali and female adult mortality from 89 per 1000 in Province 1 to 159 in Sudurpashchim. Similarly, male life expectancy is between 64.9 years in Karnali and 71.8 years in Madhesh and female male life expectancy between 69.6 years in Sudurpashchim and 77.0 years in Province 1. Mountain ecological belt and Sudurpashchim and Karnali provinces have high mortality and high poverty levels, whereas Terai and Hill ecological belts and Province 1, Madhesh, and Bagmati and Gandaki provinces have low mortality and poverty levels.

**Conclusions:**

This is the first use of CRVS system data in Nepal to estimate national and subnational mortality levels and differentials. The national results are plausible when compared with Global Burden of Disease and United Nations World Population Prospects estimates. Understanding of the reasons for inequalities in mortality in Nepal should focus on improving cause of death data and further strengthening CRVS data.

**Supplementary Information:**

The online version contains supplementary material available at 10.1186/s12889-022-14638-z.

## Background

Nepal is a relatively poor country, whose population of approximately 30 million has significant socio-economic disparities and reside in areas of considerable geographic diversity, from remote and mountainous regions to densely-populated cities [1–3]. Like other South Asian countries, Nepal is experiencing an epidemiological transition from high levels of child mortality and infectious disease mortality to the increasing importance of adult and non-communicable disease (NCD) mortality driven by rapid life-style change, unhealthy diet, tobacco use, alcohol consumption and reduced physical activity [4, 5]. It also recently in 2015 experienced a major earthquake, which resulted in the deaths of 9000 people [6]. Reliable and timely mortality data are therefore essential to know the level, trends and differentials of key mortality indicators, including adult mortality and life expectancy at birth, which provide evidence to health policymakers to monitor the progress of national and international health goals, including the Sustainable Development Goals (SDGs) [7–10].

Despite this need for reliable mortality indicators, not much is known about mortality levels and differentials in Nepal. A high-quality civil registration and vital statistics (CRVS) system is the optimal source of routine data to provide mortality statistics, with seven out of 17 SDGs best measured using data from a CRVS system [11, 12]. However, despite many decades of implementation of the CRVS system, national authorities have not previously used death registration data from the CRVS system to make estimates of mortality indicators. A study conducted to assess the quality and completeness of death registration data found that the completeness of the paper-based death registration system (i.e. offline data) is 69% at the national level, being below 50% in recent years in Madhesh and Karnali [13]. The recently introduced online death registration system still only operates in a minority of districts throughout the country, and so has completeness of just 32% [13]. Further, a limitation of the offline registration data for the production of mortality indicators is that it does not provide aggregated data by age at death. Further detail of the Nepal CRVS system is provided elsewhere [13].

In the absence of reliable data sources, the primary estimates of mortality in Nepal are reliant on irregular data from Population Censuses (conducted every 10 years, most recently in 2011) and surveys such as the Demographic and Health Survey (DHS) and the CRVS Survey [14–16]. The most recent data of reported deaths at all ages from one of these sources is from the CRVS Survey 2015/16, which had an estimated completeness of 75% [13]. Official mortality estimates are made indirectly by the Central Bureau of Statistics (CBS), who estimate the level and patterns of child mortality, life expectancy at birth and maternal mortality ratio by applying demographic methods to different data sources, including the Population Census and DHS [17, 18]. International groups such as the Global Burden of Disease (GBD) and United Nations World Population Prospects (UNWPP) also estimate mortality indicators of Nepal, at the national level only, based on limited data and large reliance on demographic and statistical modelling, including estimates from other countries [13, 19, 20]. This has resulted in significant variation in the estimates. For instance, between 1981 and 2011, CBS estimated female life expectancy in Nepal increased from 48.1 to 68.0 years and male life expectancy from 50.9 to 65.4 years [18]. Similarly, from 1980 to 85 to 2015–20 UNWPP estimated female life expectancy increased from 48.9 to 71.7 years and male life expectancy increased from 48.1 years to 68.8 years, while GBD estimated female life expectancy increased from 59.1 to 73.3 years and male life expectancy 57.7 to 68.7 years respectively between 1990 to 2017 [4, 21].

In Nepal, there is a lack of mortality indicators at the subnational level, despite increased responsibilities for the new provinces for health program development and policy making. Nepal’s seven provinces and 753 municipalities and rural municipalities were created in 2015 after historic political change following the abolition of the monarchy. The Constitution of Nepal 2015 made the provision that the access to basic health services is a fundamental human right for every citizen, which has been implemented via the promulgation of National Health Policy of 2014, five-year Nepal Health Sector Strategy (2015–2020) and Second Long Term Health Plan (1997–2017) [22–25]. These policy documents envision universal health coverage by means of equitable resource allocation and investment, and so require subnational data to inform local health policy development. There are likely to be considerable differences in mortality indicators between these provinces due to Nepal’s socio-economic disparities. There is a substantial literature on how mortality is associated with different aspects of human development, for example a strong association between socioeconomic status, including family income, and mortality, while income inequality within the population is also correlated with mortality levels [26–31].

Although a complete and well-functioning CRVS system is the gold standard for generating mortality data to measure national and subnational mortality indicators, incomplete death registration can be used to generate mortality indicators using different demographic methods, as used by the UN WPP and GBD in many countries [4, 21]. Given the policy need for reliable and disaggregated information on mortality levels and trends, as well as the opportunity to better exploit data from the existing, if imperfect, CRVS system in the country, we believe it is imperative to investigate how death registration data and other mortality data sources can be collectively used to estimate levels and subnational differentials in mortality across all ages in Nepal, which has never been conducted before. The first objective of this paper is to estimate levels and subnational differentials in mortality across all ages in Nepal, based on application of a method to derive mortality estimates from incomplete data sources. The second objective is to assess the plausibility of the subnational mortality estimates by assessing the association with monetary poverty headcount rates.

## Methods

### Data sources

We used the offline (paper-based) death registration system and CRVS Survey as data sources for deaths by sex and geography. The online death registration system was not used because of its low completeness, only capturing one-third of deaths, while the Population Census of 2011 was conducted too far in the past compared with the other data sources to be considered for inclusion. We analysed 5 years (2013–2017) of offline death registration data obtained from the Department of National ID and Civil Registration (DONIDCR) [19, 32, 33]. The nationally representative CRVS Survey of 2015/16 collected information on different aspects of the CRVS system in Nepal. This survey, conducted among 80,000 sampled households, asked respondents to report on deaths in their household in the previous 3 years. In total, there were 4532 deaths reported, resulting in an estimated 306,073 deaths at the national level during the recall period [16]. All deaths data were converted proportionally from the Nepali calendar (which commences in April) to the Gregorian calendar.

### Completeness assessment

We used estimates of completeness of both offline and CRVS survey registered/ reported deaths nationally, for each of the seven provinces and each of the three ecological belts, made in a separate study using the empirical completeness method [13]. The empirical completeness method was developed from approximately 2500 country-years of data from over 100 countries and estimates completeness by sex based on the key drivers of the crude death rate (CDR - deaths divided by population multiplied by 1000) - it is suitable for estimating both national and subnational completeness using routine death registration data [34]. The inputs into the model were the registered/reported crude death rate (per 1000 population), estimated under five mortality rate, and percentage of the population the population aged 65 years (Additional File 1). Further details on the application of the empirical completeness method for Nepal and more generally is described elsewhere [13, 34]. National offline death registration completeness over the period ranged from 62.0 to 71.1% and was consistently higher for males than females (Additional File 2: Tables A1-A2). Subnationally, death registration completeness varied, and was consistently lower in Karnali province and Madhesh [13]. The completeness of CRVS survey reported deaths was 54.4% for 2014 and 74.9% for 2015; the lower completeness in 2014 appears to be due to issues with recalling respondents’ deaths further in the past. Estimates were not made for some earthquake affected areas, including Mountain and Hill ecological belts and Bagmati province for 2015, due to issues with accurately estimating completeness when there is a mortality shock such as this [6, 13].

### All-cause mortality statistics

A drawback of the offline registration data is that there is no age at death data for which to calculate all-cause mortality statistics, including under-five mortality (*5q0* – the risk of a child dying from live birth to 5 years of age per 1000 live births), adult mortality (*45q15* – the probability of dying between 15 and 60 years per 1000 population), and life expectancy at birth (e_0-_ the average number of years that a newborn could expect to live if the current death rates do not change) for each province and ecological belt. Given that the offline registration data, of the data sources available, are available for the greatest number of years, this issue needed to be addressed. We therefore adopted an extension of the empirical completeness (the equivalent deaths method) to generate complete life tables and hence estimated adult mortality and life expectancy at birth at the national and subnational levels [35]. The method extension relies on estimation of the true number of deaths by taking registered or reported deaths (by sex) and dividing by the estimated all-age completeness (also by sex, estimated using the empirical completeness method). Using age- and sex-specific population data, an estimate of under-five mortality (*5q0*; which is also used in the calculation of all-age completeness) (see Additional File 1) and a model life table, we can iteratively change adult mortality (*45q15*) until it provides age-specific mortality rates that, combined with age-specific population data, are equivalent to the estimated true number of deaths calculated above. The model life table employed is the Model Life Table with Flexible Standards (MLTFS), with the GBD life table for Nepal used as the standard.

The conventional method to generate life tables from incomplete registration data uses an estimate of under-five mortality and the adult mortality adjusted for completeness of registration at ages 5 years and above, which are then inputted into a model life table; however, use of this method is not possible given there are no age at death data in the offline registration data [35]. A limitation of the empirical completeness method extension is that it can be biased by inaccurate age-specific population data, which is used in converting the life table to an estimate of total deaths. Further details of the empirical completeness method extension is provided elsewhere [35].

**Fig. 1:**
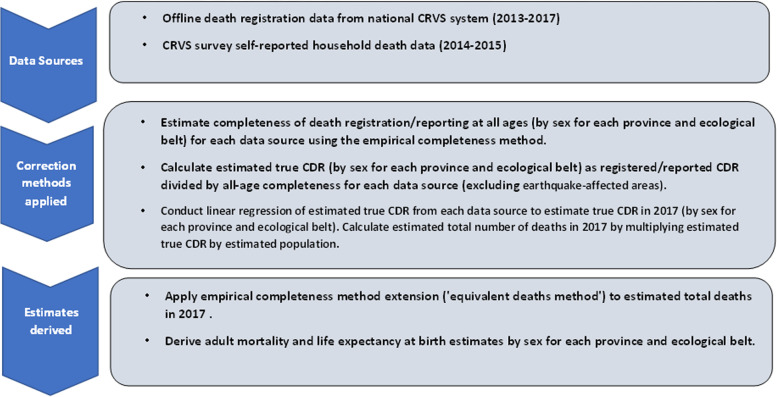
Summary of methodology used to estimate subnational all-cause mortality of Nepal 2017

To estimate the true number of deaths in 2017, we calculated the estimated true CDR (registered/reported deaths divided by all-age completeness) for all years for which we have completeness estimates (offline registration 2013–17, CRVS Survey 2015), except for those for subnational areas affected by the earthquake as well as 2014 estimates for the CRVS Survey (which are affected by recall bias) [13]. We used linear regression of the estimated true CDR of each source-year for each sex in each subnational area, with the resultant coefficient for the variable of year used to estimate the true CDR in 2017, which was converted using population data to the estimated true number of deaths. Estimated deaths for 2017 by sex and subnational levels were used in the empirical completeness method extension to estimate adult mortality and life expectancy at birth. The advantage of modelling trends in estimated true CDR is that it reduces bias introduced by the offline registration data that only have date of registration available, and not date of occurrence. The population estimates used in the analyses were projections of the population made using the Population Census 2011 data (see Additional File 1). A graphical summary of methodology adopted for the all-cause mortality estimation is presented in Fig. 1.

### Plausibility assessment of mortality estimates

The estimated mortality indicators were assessed for plausibility by comparing them with subnational poverty headcount rates, given the literature presented above demonstrating their relationship. Direct estimates of national and subnational monetary poverty headcount rates were based on the Nepal Living Standard Survey 2010/11 [36, 37]. Although the multidimensional poverty index (MPI) has also been estimated and published by the National Planning Commission, Nepal based on the Nepal Multiple Indicator Cluster Survey (MICS) 2014, we used monetary poverty because child mortality, one of our outcomes, is one of the indicators used for MPI estimation and because the MPI is not available for ecological belts [38]. The monetary poverty headcount rate is based on the Cost of Basic Needs (CBN) approach and is estimated as the percentage of people living below the poverty line [36]. The poverty line was defined as the expenditure value required by an individual to fulfill his/her basic requirements on food and non-food items. More specifically, the poverty line was constructed based on the individual’s expenditure for their daily calorie requirement (2220 kcal per day) [36]. There were no more recent estimates of the poverty headcount rate in Nepal. Mortality versus poverty estimates is plotted separately by sex and subnational levels, and the degree of the association is measured by the R-squared.

## Results

### All-cause mortality statistics for 2017

The major all-cause mortality indicators, including under-five mortality, adult mortality and life expectancy at birth are presented by sex and geographic areas in Table 1. Estimated national and subnational poverty headcount ratios, registered/reported CDR and estimated true CDR by ecological belts and provinces are also provided in the same table. Registered/reported CDR from all sources is provided in Additional File 2: Tables A3-A4, estimated true CDR is shown in Additional File 2: Table A5, while Additional File 2: Figs. A1 and A2 show the estimated true CDR from 2013 to 17 and modelled estimate of true CDR in 2017 for each province and ecological belt.

**Table 1:** Estimated (true) CDR^a^, registered CDR, projected population, annual deaths, under-five mortality, adult mortality, life expectancy at birth by sex and poverty headcount rate, Nepal 2017

Area	Estimated true CDR (per 1000 population)		Registered CDR (per 1000 population)		Projected Population 2017 (number)		Estimated annual deaths 2017 (number)		Under-five mortality^b^, per 1000 live births		Adult mortality, per 1000 population		Life expectancy at birth (years)		Poverty headcount rate (%)
	Male	Female			Male	Female	Male	Female	Male	Female	Male	Female	Male	Female	
**Nepal**	**6.61**	**4.80**	**4.86**	**3.14**	**13,969,014**	**14,841,549**	**92,309**	**71,247**	**35.6**	**31.1**	**159**	**116**	**69.7**	**73.9**	**25.2**
***Ecological Belts***															
Mountain	8.30	6.59	6.05	3.57	907,769	959,042	7533	6320	51.8	47.7	181	140	67.3	70.9	42.3
Hill	7.00	4.83	5.65	3.61	5,814,237	6,327,475	40,714	30,569	32.8	27.2	154	101	70.3	75.6	23.5
Terai	6.09	4.38	4.08	2.69	7,247,009	7,555,031	44,125	33,056	34.8	31.3	148	106	70.3	74.6	23.4
***Provinces***															
Province 1	6.51	4.29	4.92	3.03	2,284,395	2,525,664	14,872	10,830	29.4	22.7	134	89	71.7	77.0	16.7
Madhesh	5.21	3.95	3.03	2.21	3,000,718	2,963,394	15,628	11,713	31.2	30.6	129	98	71.8	75.2	26.7
Bagmati	6.07	4.56	4.71	3.41	3,062,868	3,063,284	18,593	13,981	27.6	23.2	138	92	71.7	76.7	20.6
Gandaki	8.08	5.28	7.63	4.79	1,130,528	1,352,744	9137	7142	25.4	21.2	144	97	71.5	76.5	21.0
Lumbini	7.28	4.96	5.91	3.78	2,312,468	2,584,405	16,833	12,810	38.9	34.3	173	124	68.7	73.0	25.3
Karnali	7.41	4.74	3.17	1.73	847,971	879,962	6283	4173	54.3	44.6	224	139	64.9	71.1	38.6
Sudurpashchim	8.08	6.38	6.10	2.82	1,330,068	1,472,096	10,742	9394	52.3	47.5	218	159	65.4	69.6	45.6

^a^Estimated by using linear regression, with the estimated true CDR as the dependent variable and year (2013–2017) as the independent variable

^b^Under-five mortality rate included in calculation of all-age completeness

### Under five mortality rate

The national under five mortality rate for 2017 as per UN IGME estimates was 35.6 per 1000 live births for males and 31.1 for females [39]. The under-five mortality rate by ecological region was highest in Mountain (male 51.8 and females 47.7), followed by Terai (male 34.8, female 31.3) and Hill (male 32.8, female 27.2). Provincial male under five mortality was highest in Karnali (54.3), and lowest in Gandaki (25.4). Similarly, provincial female under five mortality was highest in Sudurpashchim (47.5) and lowest in Gandaki (21.2). A map of provincial variation in under five mortality by province and sex is presented in Additional File 2: Fig. A3 and shows it to be highest in the western part of Nepal.

### Adult mortality

The estimated adult mortality (per 1000 population) was 159 for males and 116 for females in Nepal for 2017. Ecologically, the estimated adult mortality was highest in Mountain (male 181, female 140), followed by Hill (male 154, female 101) and Terai (male 148, female 106). By province, estimated male adult mortality was highest in Karnali (224) and Sudurpashchim (218), and lowest in Bagmati (138), Province 1 (134) and Madhesh (129). Female adult mortality was also highest in Sudurpashchim (159), followed by Karnali (139) and Lumbini (124), and lowest in Madhesh (98), Gandaki (97) and Bagmati (92) and Province 1 (89). The geographic variation in adult mortality level by province and sex is shown in Additional File 2: Fig. A4, with western Nepal again having highest adult mortality while eastern Nepal’s mortality being lower overall.

### Life expectancy at birth

**Fig. 2:**
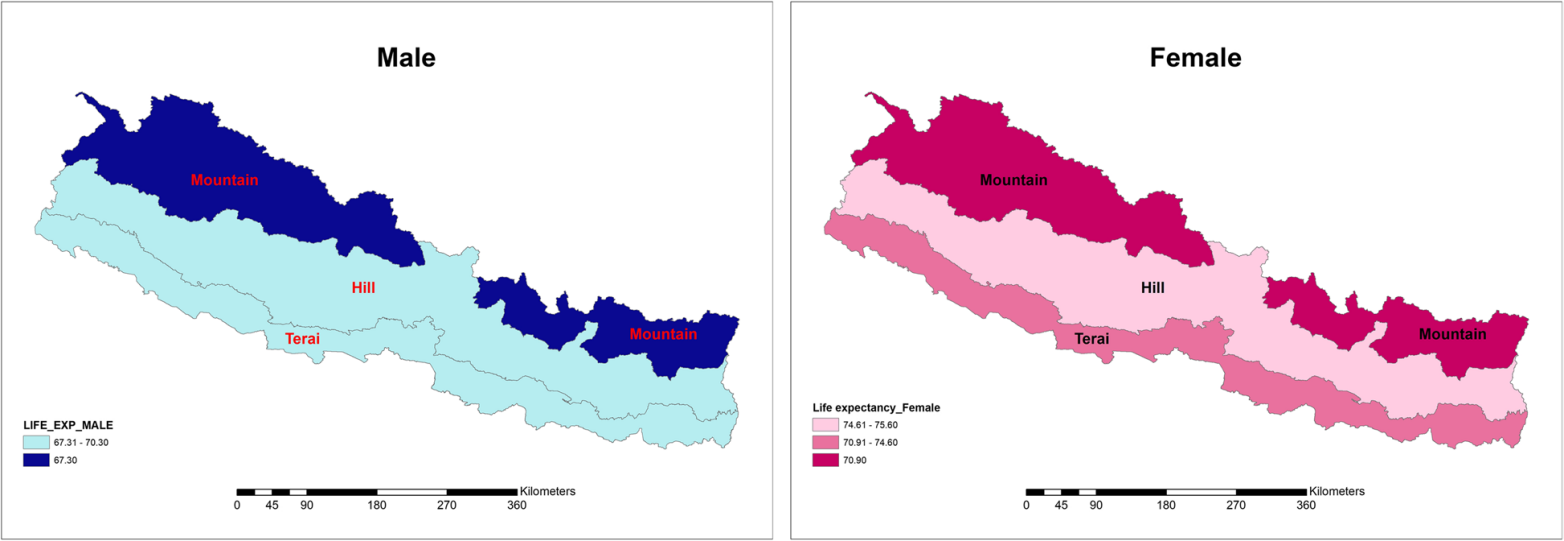
Life expectancy at birth by ecological belts and sex, Nepal, 2017

National life expectancy at birth in 2017 was estimated 69.7 years for males and 73.9 years for females. In ecological belts, there was equal male life expectancy in Hill and Terai (70.3 years), where female life expectancy was 75.6 years and 74.6 years, respectively. Mountain region has clearly the lowest life expectancy for each sex (male 67.3 years and female 70.9 years). Maps of life expectancy by ecological belt are shown in Fig. 2.

**Fig. 3:**
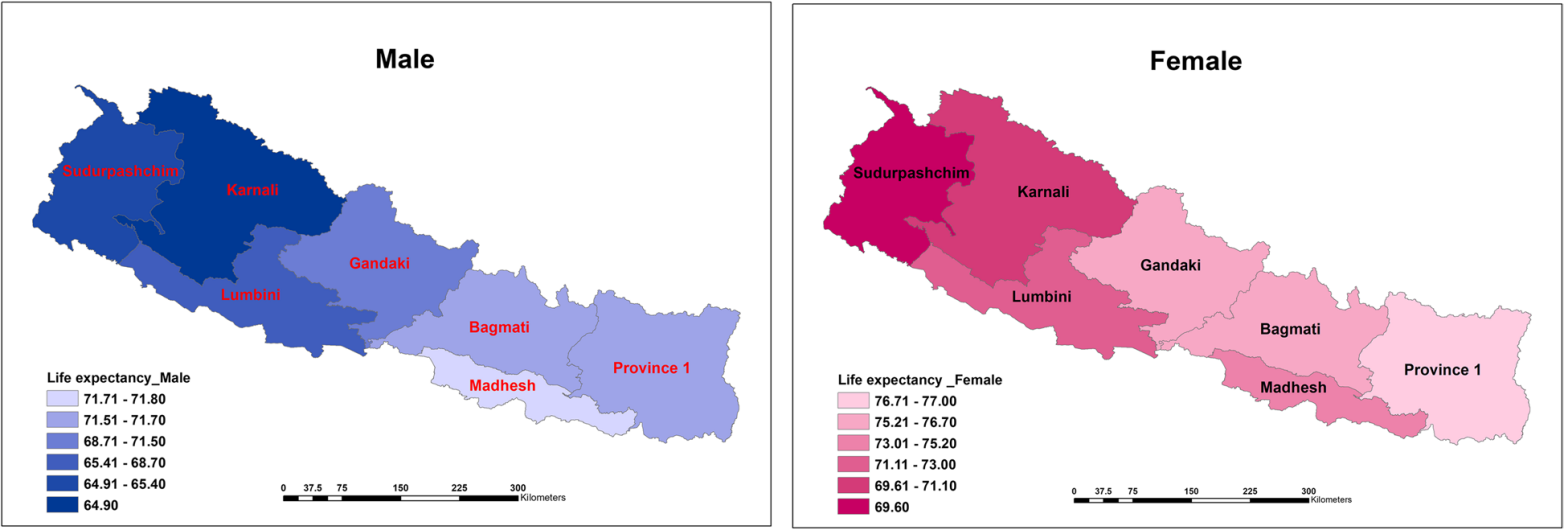
Life expectancy at birth by provinces and sex, Nepal, 2017

Figure 3 presents the geographic variations in estimated life expectancy at birth in Nepal by province and sex in 2017. Male life expectancy was highest in Madhesh (71.8 years), Province 1 and Bagmati (71.7), and Gandaki (71.5), and lowest in Sudurpashchim (65.4) and Karnali (64.9). For females, life expectancy was highest in Province 1 (77.0) followed by Bagmati (76.7), Gandaki (76.5), while again lowest in Karnali (71.1) and Sudurpashchim (69.6). Again, a clear pattern of lower life expectancy (higher mortality) is found in the western part of Nepal.

### Subnational mortality compared with monetary poverty

**Fig. 4:**
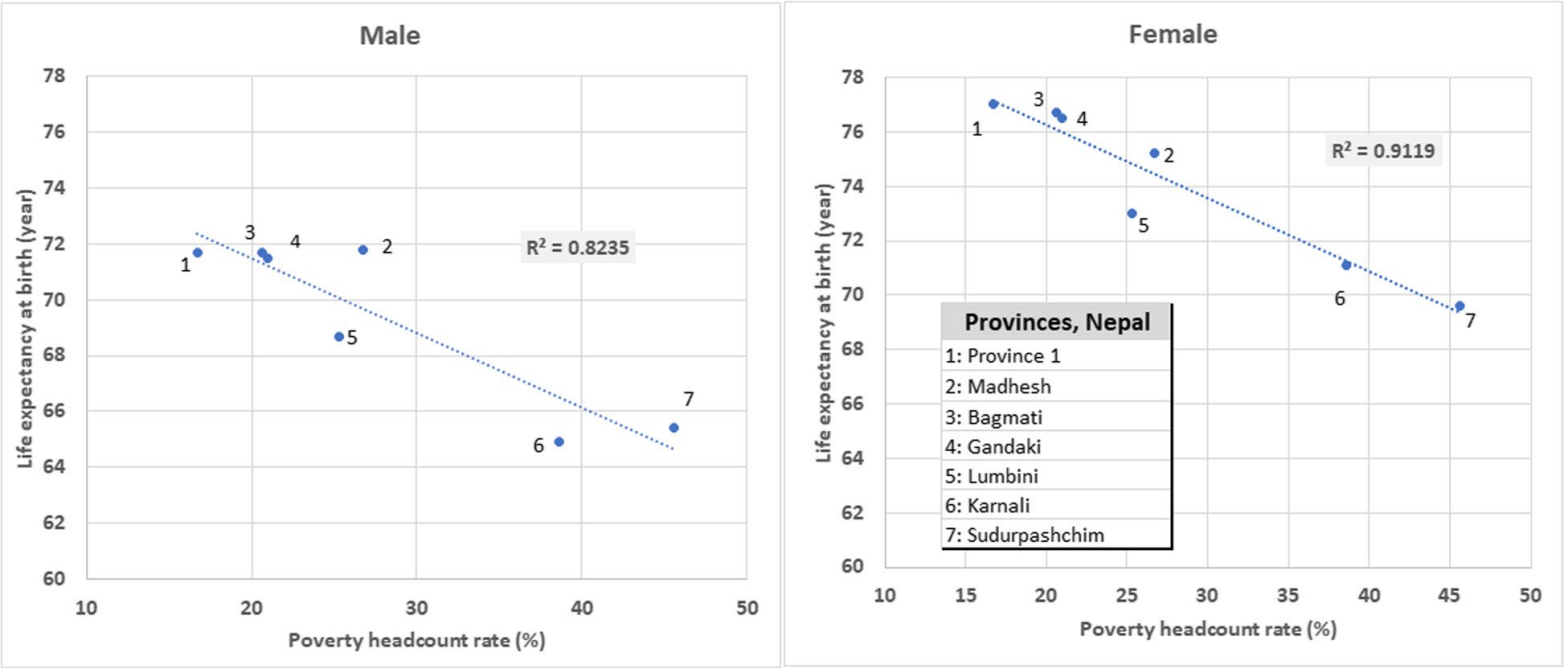
Life expectancy at birth and poverty headcount rate by provinces and sex, Nepal, 2017

**Fig. 5:**
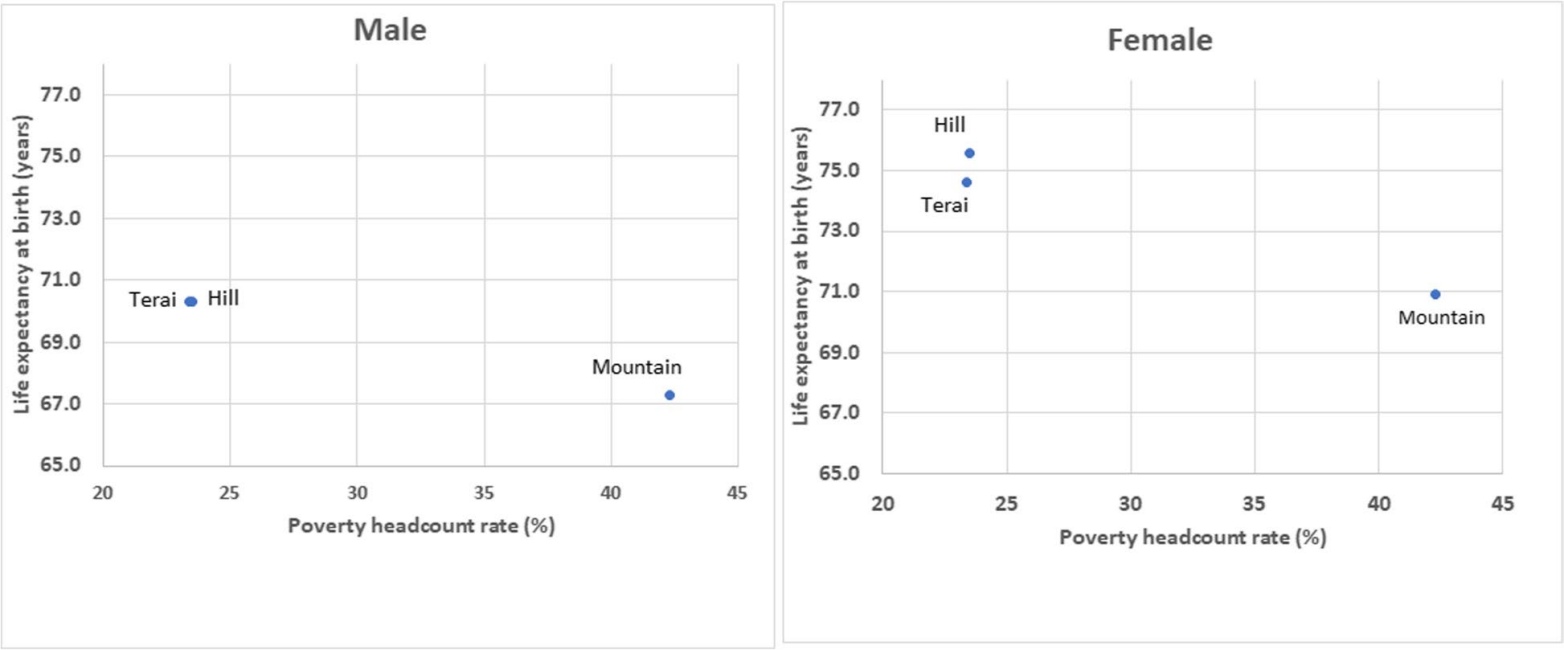
Life expectancy and poverty headcount rate by ecological belts and sex, Nepal, 2017

The provincial estimates of the monetary poverty based on the Nepal Living Standard Survey 2010/11 (poverty headcount rate) are shown in Table 1. Nationally 25.2% of people were living below the poverty line. We found a strong positive association for both sub-national domains (provinces and ecological belts) between the poverty headcount rate and under-five mortality and adult mortality, and a strong negative association with life expectancy. (Figs. 4 and 5, Additional File 2: Figs. A5-A8) Karnali and Sudurpashchim had the lowest life expectancy and the highest poverty headcount rate, while Province 1, Bagmati and Gandaki both had the highest male female life expectancy and lowest poverty headcount rate. Madhesh also had a high male life expectancy but not as low poverty rate, and its male life expectancy was highest when compared with the fitted linear relationship (i.e. what the poverty headcount would suggest). The R-squared of the relationship with life expectancy is 0.824 for males and 0.912 for females. The relationship is also strong for adult mortality (R-squared 0.804 males, 0.894 females) and under-five mortality (R-squared 0.848 males, 0.923 females).

Out of all three ecological belts, Mountain region had the highest level of mortality levels (lowest life expectancy) and highest headcount poverty rate (Fig. 5, Additional File 2: Figs. A7-A8). Hill and Terai had almost similar under-five and adult mortality rates for each sex and have very similar life expectancy for both male and females; compared with Mountain these mortality rates are much lower (life expectancy higher) and the headcount poverty rate is much lower.

## Discussion

This study has estimated three major mortality indicators for Nepal – under-five mortality, adult mortality, and life expectancy – by ecological belts and provinces by analysing the trend of five-year death completeness-adjusted registration and reporting data. The findings show considerable differences in these mortality indicators by province, with the western provinces of Lumbini, Karnali and Sudurpashchim and the ecological belt of Mountain having high mortality and low life expectancy compared with the other areas of the country. We also assessed the plausibility of estimated mortality indicators against the estimated monetary poverty levels in those sub-national regions and found a strong association between them, as expected. This offers some empirical support for the relative pattern of mortality across the country that we have identified.

These indicators are consistent with those made by the GBD and UNWPP. Our estimated adult mortality rates (male 159 per 1000, female 116) are lower than the UN (male 171, female 133), and GBD (male 180, female 130) estimates [4, 21]. Another source of adult mortality is sibling survival data in the DHS 2016, however the only published estimates from these data are for ages 15–49 years; further, there are methodological challenges of this method, including inherent survival and recall bias and the issue of underreporting [5, 15, 40]. Our national-level estimate of life expectancy at birth are 69.7 for males and 73.9 for females; male estimates are slightly higher than both UNWPP (68.8) and GBD (68.7) estimates and moderately higher than the UNWPP estimate (71.7) but very close to the GBD estimate (73.3) [4, 21]. Higher life expectancy and lower mortality estimated by this study is consistent with higher completeness of registration/reporting compared with UNWPP and GBD estimates [13]. Our estimates are significantly higher than 2011 Population Census-based estimates (male 65.4 and female: 68.0), although the difference would be partly explained by the increase in life expectancy in recent years [3]. The similarity of our estimates to the UNWPP and GBD provides reassurance in their plausibility given the use of incomplete registration and survey data and supports the extension of the empirical completeness method that uses the age pattern of deaths from a model life table [36].

Our analysis highlights major differences in adult mortality rates by province, with the highest provincial rate being 74% higher than the lowest rate for males and 78% higher than the lowest rate for males. The poverty rates in Karnali and Sudurpashchim provinces (more than double Province 1) and Mountain belt (almost double Hill and Terai) are all high and correspond with their high mortality/low life expectancy, while Lumbini has a more moderate poverty rate and high mortality/low life expectancy. The close association of our mortality estimates with poverty levels is justified by existing literature about the contribution of higher level-of socio-economic development and lower level of poverty for reducing mortality levels [29, 41]. Findings consistent with ours have been found by some studies regarding tobacco consumption and other risk factors associated with adult mortality in those subnational areas. For example, a nationally representative study among adults aged 15–49 years found highest male (40%) and female (13%) smoking prevalence in Sudurpashchim and Karnali provinces and the smoking prevalence rate was also found highest in mountain region for both sexes (male 32%, female 9%) [42]. Likewise, the Nepal Multiple Indicator Cluster Survey 2019 also found that the highest proportion (15%) of females that had ever consumed any tobacco product was in Karnali, while in Sudurpaschim 60% of males had ever consumed tobacco, the second-highest of all provinces [43].

Moreover, the subnational results are also consistent with the DHS 2016 results showing the percentage of women aged 15–49 years who state that distance to a health facility is a serious problem they face in accessing health care when they are sick. Distance is more an issue in Karnali (72%), Sudurpashchim (70%), and Mountain (66%), where life expectancy is lower, but is less a problem in in Gandaki (38%), Bagmati (43%), Province 1 (50%) and Hill region (51%) where life expectancy is higher [15]. These results also reflect the difference and difficulties in accessing health services due to different terrain, climate and transport access between provinces and ecological belts [44]. These findings inform the need to develop interventions to mitigate high levels of smoking in these areas, a major risk factor for chronic respiratory diseases, lung cancer and cardiovascular diseases, to improve access the primary health care, as well as broader efforts to address the structural reasons for higher poverty. Policymakers need to pay close attention to the higher levels of adult mortality, in particular, because they are the most economically active age group, and can be also more vulnerable to both communicable (including HIV/AIDS, Tuberculosis) and non-communicable diseases [5, 45]. More generally, national, and local governments should focus on health interventions to reduce the mortality burden in these areas.

To our knowledge, this is the first study in Nepal to estimate the subnational mortality differences using routine death registration data. The results presented provide evidence of mortality differentials in Nepal that can be used to assist health policy and that would also be more reliable if there were a complete CRVS system. An improved CRVS system would be able to provide continuous, reliable, and disaggregated mortality statistics to generate evidence for policy interventions and to monitor the progress of SDGs and other health indicators. CRVS data is especially beneficial subnationally, and in Nepal the newly formed provinces are currently dependent on poor quality or non-existent data and are designing local policies without reliable evidence. Improving the CRVS system will have major benefits. Firstly, a large volume of information related to the personal events has been stored at each local level in event registers and notification forms for decades and this could be used to produce reliable mortality statistics at local level. Secondly, the locally produced results could be easy validated and evaluated to ensure their quality on regular basis. Thirdly, local governments can produce and use those statistics as per their specific requirements. This practice will also help to build a system to utilize CRVS system generate vital statistics in local level policies.

Nepal also lacks reliable cause of death data to guide and monitor effective policy interventions and equitable allocation of the resources. Cause of death data are not adequately reported in the national CRVS system and hospital deaths also either do not record a cause of death or use a medical certificate of cause of death that is not aligned with the World Health Organization International Medical Certificate of Cause of Death using the International Classification of Diseases (ICD-10) [46, 47]. More than 70% of deaths in Nepal occur in the community where only a rudimentary list of causes like ‘natural deaths’ are collected in census and surveys which have very limited statistical utility, and with no routine cause of death data collection [48, 49]. In this context, modelled global estimates including GBD are only the present alternate source of COD in Nepal [4].

This study has some limitations that are related to the drawbacks of available mortality data in Nepal. Firstly, to be able to generate age-specific mortality statistics given no age at death in death registration data, we relied on the use of a model life table. The plausibility of resultant national adult mortality and life expectancy estimates with both the GBD and UN however suggests that the choice of model life table is appropriate. Additionally, the empirical completeness method extension employed to integrate the model life table with the empirical completeness method can produce estimates biased by incorrect population data, especially given that a projection from the 2011 Population Census was used. Such an analysis should be repeated once the 2021 Census population figures are available. The under-five mortality used was also an estimate, however it is based on multiple sources of data for each subnational area and scaled to IGME national estimates.

## Conclusion

Reliable evidence of mortality levels and differentials can help to design policy for reducing the number of premature deaths in a population. Although Nepal lacks a high quality CRVS system to provide such data, this study has been able to use demographic methods to generate mortality estimates, nationally and sub nationally, based on incomplete death registration data that has no data on age at death. The results provide plausible estimates of adult mortality and life expectancy at the national level. We believe this study also contributes to Nepal’s CRVS strengthening efforts by manifesting the utility of the vital statistics component of the CRVS system as a source of important demographic and health indicators. Further, the study is empirical, in the sense that we used routine registration data for estimating important mortality indicators, including SDGs, in place of more commonly used census and survey data, which produce irregular data and are subject to uncertainty in the limited mortality data they collect which commonly require the use of indirect methods. Ultimately, the subnational mortality estimates made in this study fulfil the existing mortality data gap to some extent and contribute as an evidence in local level health policy interventions in Nepal. Further understanding of the reasons for inequalities in mortality in Nepal should focus on improving cause of death data, including interventions for integrating both hospital and community deaths via systematic verbal autopsy with the routine CRVS system to generate cause-specific mortality statistics on regular basis [50]. Also, to obtain more timely and reliable data, the online registration system needs to be further strengthened and scaled up to all parts of the country.

## Supplementary Information

**Additional file 1.**

**Additional file 2: Table A1.** Summary of All-Age Completeness (%), Offline Registered Deaths (2013–2017) []. Table A2. Online death registration completeness (%) by area and sex, Nepal (2017–2019) []. **Table A3.** Reported CDR (per 1000) by subnational levels and sex, CRVS Survey 2015. **Table A4.** Registered/reported CDR (per 1000) by sex and subnational level, offline death registration data (2013–2017). **Table A5.** Estimated true CDR (per 1000) by national and subnational levels and sex, based on offline death registration data (2013–2017) and CRVS Survey (2015) data adjusted for completeness. **Fig. A1.** Estimated true CDR (per 1000) by Ecological Belts (2013–2017), and modelled estimated true CDR (2017). **Fig. A2.** Estimated true CDR (per 1000) by Provinces (2013–2017), and modelled estimated true CDR (2017). **Fig. A3.** Under-five mortality (*5q0*) by provinces and sex, Nepal, 2017. **Fig. A4.** Adult Mortality (45q15) by Provinces and Sex, Nepal, 2017. **Fig. A5.** Under-five mortality (per 1000) and poverty headcount rate (%) by provinces and sex, Nepal, 2017. **Fig. A6.** Adult mortality (per 1000) and poverty headcount rate (%) by provinces and sex, Nepal, 2017. **Fig. A7.** Under-five mortality (per 1000) and poverty headcount rate (%) by ecological belts and sex, Nepal, 2017. **Fig. A8.** Adult mortality (per 1000) and poverty headcount rate (%) by ecological belts and sex, Nepal, 2017.

## Data Availability

Request for data access should be directed to the corresponding authors and will be granted subject to approval by the Department of NID and Civil Registration and Central Bureau of Statistics, Nepal.

## Abbreviations

CBS: Central Bureau of Statistics
CBN: Cost of Basic Needs
CDR: Crude Death Rate
CRVS: Civil Registration and Vital Statistics
DHS: Demographic and Health Survey
DONIDCR: Department of Civil Registration and Civil Registration
GBD: Global Burden of Disease
IGME: Inter-agency Group for Child Mortality Estimation
MICS: Multiple Indicator Cluster Survey
MIS: Management Information System
MLTFS: Model Life Table with Flexible Standards
SDG: Sustainable Development Goals
UN: United Nations
UNDP: United Nations Development Programme
UN WPP: United Nations World Population Prospects
VA: Verbal Autopsy
