# Supplementary material for: Estimation of national and subnational all-cause mortality indicators in Nepal, 2017
Source: BMC Public Health. 2022 Dec 3;22:2262. doi: 10.1186/s12889-022-14638-z (PMC9719662; doi:10.1186/s12889-022-14638-z)
Supplement: Supplementary file 1 — Additional file 1. [file 12889_2022_14638_MOESM1_ESM.docx]

**Additional File 1**

**Population projection**

Projected population by age-sex and by 3 ecological belts and seven provinces was used as a denominator for estimating different mortality indicators and as an input in both of completeness models. Population by age, sex and province/ecological zone was estimated from the 2011 Population Census and official population projections from 2011-2031. (1) Population projections are available for 2016 and 2021 by age and sex at the national level, and for 2016 and 2021 by sex at the national and subnational level. At the national level, we interpolated total population by calculating the exponential growth rates for 2011-16 and 2016-21, and for each age using linear interpolation of the proportion of the population at that age using the same time periods. (2) At the subnational level, we also interpolated total population using the exponential growth rates for 2011-16 and 2016-21 and estimated population for each age using the ratio of the proportion of the population at that age in the subnational area compared with at the national level; we adjusted the final estimates to ensure the sum of the population at all ages equaled the estimated total population.

**Under five mortality (*5q0*) estimation**

We used the *5q0* estimates from the United Nations Inter-agency Group for Mortality Estimation (IGME) at the national level, estimated by trend analysis of different available sources. *5q0* is estimated for each province and ecological belt by using census 2011 children survival data and Demographic and Health survey (DHS)-2016 birth history data.(3) Further, the average of census and DHS generated *5q0* was calculated and its ratio to the national estimate of 5q0 from both sources was again calculated. This was used to scale subnational 5q0 to the national estimate of IGME for each year.(3)

**Data adjustment for earthquake 2015 affected areas**

A Post-Earthquake survey was conducted in 31 affected districts by CBS Nepal in 2016-2017, with the primary objective of assessing the damage of the housing units due to earthquake happened in 2015 and collected deaths due to earthquake. It showed that the earthquake killed more than 9000 people in 31 districts and that a very high proportion of the earthquake deaths occurred in Bagmati province (90%), while all deaths either occurred in Mountain (49%) or Hill (51%) ecological zones. Therefore, we excluded Mountain, Hill, and Bagmati for 2015 (offline registration and CRVS Survey) and Mountain for 2016 (Mountain also had an unusually high number of registrations in 2073 (2016-17), likely due to the inclusion of delayed registrations from the previous year); see Table A4. Also, in Hill and Bagmati, the 2016 data are based on death registration for 2073 (15 April 2016- 15 April-2017), and not a combination of 2072 and 2073, because 2072 deaths were affected by the earthquake, and these cannot be disaggregated further by month. (4)

**References**

1. Central Bureau of Statistics. National Population and Housing Census 2011 Population Projection (2011-2031). Kathnamdu; 2014.

2. Shryock HS, Siegel JS, Larmon EA. The methods and materials of demography: US Bureau of the Census; 1973.

3. Pandey, S., Adair, T. 2022. Assessment of the national and subnational completeness of death registration in Nepal, *BMC Public Health*, 22: 429.

4. Central Bureau of Statistics. Household Registration for Housing Reconstruction Program (HRHRP). In: Central Bureau of Statistics, editor. Post Earthquake Survey. Kathmandu; 2017.
