# Supplementary material for: Estimation of national and subnational all-cause mortality indicators in Nepal, 2017
Source: BMC Public Health. 2022 Dec 3;22:2262. doi: 10.1186/s12889-022-14638-z (PMC9719662; doi:10.1186/s12889-022-14638-z)
Supplement: Supplementary file 2 — Additional file 2: Table A1. Summary of All-Age Completeness (%), Offline Registered Deaths (2013–2017) []. Table A2. Online death registration completeness (%) by area and sex, Nepal (2017–2019) []. Table A3. Reported CDR (per 1000) by subnational levels and sex, CRVS Survey 2015. Table A4. Registered/reported CDR (per 1000) by sex and subnational level, offline death registration data (2013–2017). Table A5. Estimated true CDR (per 1000) by national and subnational levels and sex, based on offline death registration data (2013–2017) and CRVS Survey (2015) data adjusted for completeness. Fig. A1. Estimated true CDR (per 1000) by Ecological Belts (2013–2017), and modelled estimated true CDR (2017). Fig. A2. Estimated true CDR (per 1000) by Provinces (2013–2017), and modelled estimated true CDR (2017). Fig. A3. Under-five mortality (5q0) by provinces and sex, Nepal, 2017. Fig. A4. Adult Mortality (45q15) by Provinces and Sex, Nepal, 2017. Fig. A5. Under-five mortality (per 1000) and poverty headcount rate (%) by provinces and sex, Nepal, 2017. Fig. A6. Adult mortality (per 1000) and poverty headcount rate (%) by provinces and sex, Nepal, 2017. Fig. A7. Under-five mortality (per 1000) and poverty headcount rate (%) by ecological belts and sex, Nepal, 2017. Fig. A8. Adult mortality (per 1000) and poverty headcount rate (%) by ecological belts and sex, Nepal, 2017. [file 12889_2022_14638_MOESM2_ESM.docx]

**Additional File 2**

**Additional Tables and Figures**

**Table A1: Summary of All-Age Completeness (%), Offline Registered Deaths (2013-2017) (1)**

|  | **2013** | | | **2014** | | | **2015** | | | **2016*** | | | **2017** | | |
| --- | --- | --- | --- | --- | --- | --- | --- | --- | --- | --- | --- | --- | --- | --- | --- |
| **Area** | Both | Male | Female | Both | Male | Female | Both | Male | Female | Both | Male | Female | Both | Male | Female |
| **Nepal (Census Projected Population)** | 71.1 | 76.0 | 64.2 | 70.7 | 73.7 | 66.7 | 62.0 | 66.6 | 57.5 | 67.9 | 73.6 | 61.0 | 69.4 | 73.0 | 65.4 |
| **Nepal (UN IGME population)** | 72.6 | 78.6 | 64.5 | 72.8 | 77.7 | 67.1 | 64.7 | 71.7 | 58.0 | 70.7 | 78.8 | 61.5 | 72.1 | 78.2 | 65.6 |
| **Nepal (GBD Population)** | 69.9 | 74.6 | 63.8 | 69.4 | 72.4 | 66.1 | 60.8 | 65.3 | 56.9 | 66.4 | 72.3 | 59.9 | 67.7 | 71.5 | 63.7 |
| ***Ecological Belts*** | |  |  |  |  |  |  |  |  |  |  |  |  |  |  |
| Mountain | 54.5 | 62.5 | 45.2 | 61.8 | 69.4 | 51.6 | - | - | - | - | - | - | 64.9 | 72.9 | 53.9 |
| Hill | 76.3 | 80.3 | 70.6 | 77.3 | 79.4 | 74.2 | - | - | - | 76.6 | 81.9 | 69.0 | 78.1 | 81.0 | 74.0 |
| Terai | 70.6 | 75.3 | 64.1 | 67.6 | 70.5 | 64.4 | 55.6 | 59.7 | 52.4 | 62.0 | 66.3 | 58.0 | 63.3 | 66.0 | 61.1 |
| ***Provinces*** |  |  |  |  |  |  |  |  |  |  |  |  |  |  |  |
| Province 1 | 83.9 | 86.1 | 80.1 | 84.4 | 85.9 | 81.8 | 83.4 | 85.3 | 80.6 | 82.7 | 85.6 | 78.4 | 74.6 | 77.0 | 72.7 |
| Madhesh | 59.7 | 64.0 | 55.7 | 52.8 | 55.4 | 51.7 | 28.4 | 29.4 | 30.8 | 40.2 | 41.6 | 42.1 | 54.1 | 55.9 | 54.1 |
| Bagmati | 73.9 | 76.3 | 70.3 | 72.3 | 73.2 | 70.8 | - | - | - | 78.4 | 80.2 | 75.6 | 77.1 | 78.3 | 75.2 |
| Gandaki | 83.5 | 87.2 | 77.9 | 88.8 | 90.6 | 85.3 | 85.1 | 88.2 | 80.3 | 87.0 | 90.2 | 81.6 | 90.2 | 91.6 | 87.5 |
| Lumbini | 80.0 | 83.2 | 74.7 | 79.8 | 80.4 | 78.6 | 77.3 | 81.2 | 71.9 | 75.5 | 80.6 | 68.6 | 77.7 | 80.1 | 74.6 |
| Karnali | 45.5 | 51.7 | 41.0 | 55.1 | 60.2 | 50.9 | 50.0 | 56.3 | 45.1 | 50.3 | 59.9 | 41.3 | 38.6 | 42.9 | 37.3 |
| Sudurpashchim | 68.9 | 81.5 | 48.9 | 61.6 | 73.8 | 46.4 | 52.7 | 67.0 | 37.1 | 67.5 | 83.1 | 41.0 | 63.8 | 76.8 | 45.8 |

**Table A2: Online death registration completeness (%) by area and sex, Nepal (2017-2019) (1)**

| **Area** | **2017** | | | **2018** | | | **2019** | | |
| --- | --- | --- | --- | --- | --- | --- | --- | --- | --- |
|  | **Both Sexes** | **Male** | **Female** | **Both Sexes** | **Male** | **Female** | **Both Sexes** | **Male** | **Female** |
| **Nepal (Census Projected population)** | 18.6 | 18.9 | 21.2 | 25.7 | 26.8 | 27.9 | 31.9 | 33.9 | 33.1 |
| **Nepal (UN Population)** | 19.3 | 20.1 | 21.8 | 26.7 | 29.0 | 28.3 | 33.0 | 36.8 | 33.3 |
| **Nepal (GBD Population)** | 18.3 | 18.5 | 21.3 | 24.9 | 25.8 | 27.3 | 30.5 | 32.6 | 31.8 |
| ***Ecological Belts*** | |  |  |  |  |  |  |  |  |
| Mountain | 6.3 | 6.9 | 7.3 | 7.4 | 7.9 | 8.5 | 35.0 | 39.2 | 32.9 |
| Hill | 12.0 | 11.4 | 15.2 | 12.8 | 12.2 | 16.4 | 30.1 | 31.5 | 32.1 |
| Terai | 12.4 | 11.9 | 15.9 | 13.3 | 12.7 | 17.1 | 39.9 | 42.0 | 40.7 |
| ***Provinces*** |  |  |  |  |  |  |  |  |  |
| Province 1 | 33.1 | 33.4 | 37.2 | 49.2 | 50.4 | 51.6 | 50.5 | 62.5 | 62.5 |
| Madhesh | 15.1 | 15.4 | 17.9 | 17.1 | 17.4 | 20.1 | 20.0 | 20.7 | 22.7 |
| Bagmati | 30.6 | 31.2 | 33.2 | 40.2 | 41.4 | 41.6 | 45.1 | 46.7 | 46.0 |
| Gandaki | 21.9 | 21.9 | 25.5 | 34.1 | 35.7 | 36.6 | 45.1 | 48.4 | 45.5 |
| Lumbini | 16.1 | 16.0 | 19.5 | 24.5 | 25.4 | 27.4 | 38.6 | 41.5 | 39.3 |
| Karnali | 10.9 | 10.3 | 14.7 | 12.6 | 12.1 | 16.6 | 13.2 | 12.9 | 17.1 |
| Sudurpaschim | 10.1 | 11.2 | 11.2 | 12.9 | 14.9 | 13.5 | 15.1 | 76.3 | 45.8 |

**Table A3: Reported CDR (per 1000) by subnational levels and sex, CRVS Survey 2015**

| **Area** | | **2015** | |
| --- | --- | --- | --- |
|  |  | **Male** | **Female** |
| **Nepal** | | **5.09** | **3.96** |
| ***Ecological Belts*** | |  |  |
|  | Mountain | *7.60* | *8.30* |
|  | Hill | *5.63* | *3.96* |
|  | Terai | 4.32 | 3.39 |
| ***Provinces*** | |  |  |
|  | Province 1 | 4.69 | 3.72 |
|  | Madhesh | 3.90 | 3.82 |
|  | Bagmati | *5.72* | *4.88* |
|  | Gandaki | 5.97 | 3.86 |
|  | Lumbini | 5.22 | 3.39 |
|  | Karnali | 6.71 | 4.16 |
|  | Sudurpashchim | 4.95 | 3.70 |

**Note:** 1. *Figures in italics are for earthquake-affected regions: Mountain, Hill, and province 3 (Bagmati) for 2015.*

**Table A4: Registered/reported CDR (per 1000) by sex and subnational level, offline death registration data (2013-2017)**

| **Area** | | **2013** | | **2014** | | **2015** | | **2016*** | | **2017** | |
| --- | --- | --- | --- | --- | --- | --- | --- | --- | --- | --- | --- |
|  |  | **Male** | **Female** | **Male** | **Female** | **Male** | **Female** | **Male** | **Female** | **Male** | **Female** |
| **Nepal** | | **5.43** | **3.26** | **5.13** | **3.36** | **4.43** | **2.78** | **4.98** | **2.93** | **4.86** | **3.14** |
| ***Ecological Belts*** | |  |  |  |  |  |  |  |  |  |  |
|  | Mountain | 5.35 | 3.23 | 5.89 | 3.55 | *5.01* | *2.97* | *7.71* | *3.82* | 6.05 | 3.57 |
|  | Hill | 5.80 | 3.52 | 5.63 | 3.74 | *5.21* | *3.30* | 5.57 | 3.22 | 5.65 | 3.61 |
|  | Terai | 5.14 | 3.05 | 4.62 | 3.02 | 3.72 | 2.31 | 4.16 | 2.56 | 4.08 | 2.69 |
| ***Provinces*** | |  |  |  |  |  |  |  |  |  |  |
|  | Province 1 | 6.39 | 3.78 | 6.29 | 3.89 | 6.10 | 3.74 | 6.10 | 3.51 | 4.92 | 3.03 |
|  | Madhesh | 3.82 | 2.48 | 3.17 | 2.21 | 1.44 | 1.08 | 2.18 | 1.64 | 3.03 | 2.21 |
|  | Bagmati | 4.74 | 3.21 | 4.39 | 3.21 | *3.55* | *2.61* | 4.49 | 3.17 | 4.71 | 3.41 |
|  | Gandaki | 6.79 | 3.89 | 7.52 | 4.61 | 6.87 | 4.05 | 7.28 | 4.14 | 7.63 | 4.79 |
|  | Lumbini | 6.61 | 3.99 | 6.15 | 4.25 | 6.18 | 3.68 | 6.03 | 3.41 | 5.91 | 3.78 |
|  | Karnali | 3.99 | 2.13 | 4.54 | 2.59 | 4.19 | 2.24 | 4.38 | 1.99 | 3.17 | 1.73 |
|  | Sudurpashchim | 6.55 | 2.85 | 5.70 | 2.78 | 5.26 | 2.43 | 7.02 | 2.60 | 6.10 | 2.82 |

**Note:** 1. *Figures in italics are for earthquake-affected regions: Mountain, Hill, and province 3 (Bagmati) for 2015 and Mountain region in 2016 (unexpectedly high level of registered CDR, presumably due to the existing impact of the earthquake in the next year death registrations). Estimates of completeness and true CDR are not made for these years.*

** For Hill and Bagmati province, the registered CDR for Nepali calendar 2073 is used (instead of 2016, which is the combination of two years 2072 and 2073) to minimize the impact of the earthquake caused deaths in 2072.*

**Table A5: Estimated true CDR (per 1000) by national and subnational levels and sex, based on offline death registration data (2013-2017) and CRVS Survey (2015) data adjusted for completeness**

| **Area** | | **Offline registration** | | | | | | | | | | **CRVS Survey** | |
| --- | --- | --- | --- | --- | --- | --- | --- | --- | --- | --- | --- | --- | --- |
|  |  | **2013** | | **2014** | | **2015** | | **2016** | | **2017** | | **2015** | |
|  |  | **Male** | **Female** | **Male** | **Female** | **Male** | **Female** | **Male** | **Female** | **Male** | **Female** | **Male** | **Female** |
| **Nepal** | | **7.15** | **5.08** | **6.96** | **5.04** | **6.65** | **4.83** | **6.77** | **4.8** | **6.66** | **4.8** | **6.87** | **5.22** |
| ***Ecological Belts*** | |  |  |  |  |  |  |  |  |  |  |  |  |
|  | Mountain | 8.56 | 7.15 | 8.49 | 6.88 | - | - | - | - | 8.3 | 6.38 | 6.38 | 4.78 |
|  | Hill | 7.23 | 4.99 | 7.09 | 5.03 | - | - | 6.97 | 4.75 | 6.97 | - | - | - |
|  | Terai | 6.82 | 4.76 | 6.56 | 4.68 | 6.24 | 4.41 | 6.26 | 4.42 | 6.18 | - | - | - |
| ***Provinces*** | |  |  |  |  |  |  |  |  |  |  |  |  |
|  | Province 1 | 7.41 | 4.72 | 7.31 | 4.75 | 7.15 | 4.64 | 7.12 | 4.47 | 6.39 | 4.17 | 6.37 | 4.63 |
|  | Madhesh | 5.97 | 4.45 | 5.71 | 4.28 | 4.91 | 3.5 | 5.25 | 3.88 | 5.42 | 4.08 | 5.84 | 4.89 |
|  | Bagmati | 6.22 | 4.57 | 6.00 | 4.53 | - | - | 5.94 | 4.45 | 6.01 | 4.54 | - | - |
|  | Gandaki | 7.79 | 5.00 | 8.3 | 5.40 | 7.78 | 5.04 | 8.07 | 5.07 | 8.33 | 5.48 | 7.19 | 4.94 |
|  | Lumbini | 7.95 | 5.34 | 7.64 | 5.41 | 7.61 | 5.12 | 7.49 | 4.97 | 7.38 | 5.07 | 7.18 | 5.02 |
|  | Karnali | 7.72 | 5.20 | 7.54 | 5.10 | 7.43 | 4.97 | 7.32 | 4.82 | 7.39 | 4.63 | 8.13 | 5.43 |
|  | Sudurpashchim | 8.04 | 5.82 | 7.72 | 5.98 | 7.85 | 6.54 | 8.45 | 6.35 | 7.95 | 6.16 | 7.82 | 6.18 |

**Figure A1: Estimated true CDR (per 1000) by Ecological Belts (2013-2017), and modelled estimated true CDR (2017)**


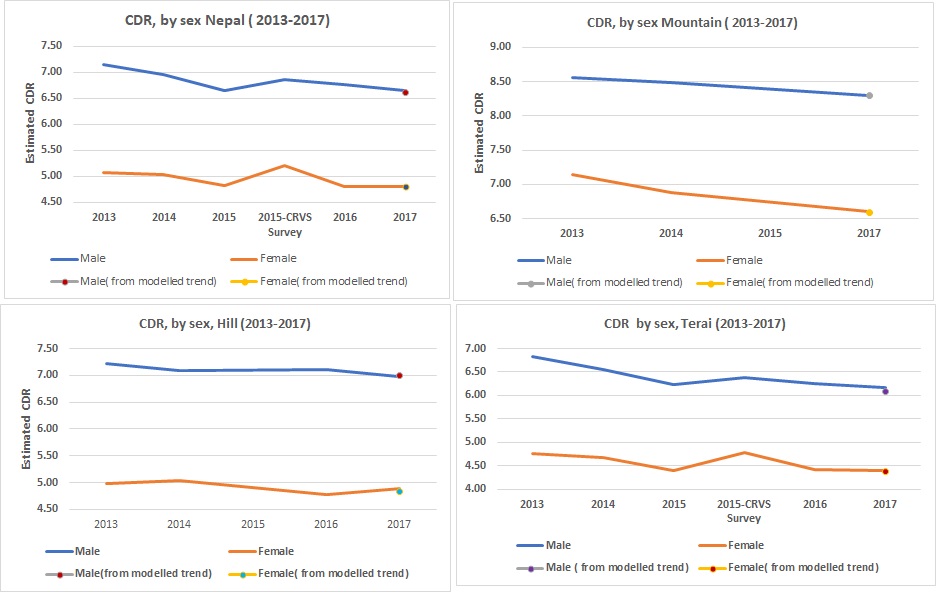


**Figure A2: Estimated true CDR (per 1000) by Provinces (2013-2017), and modelled estimated true CDR (2017)
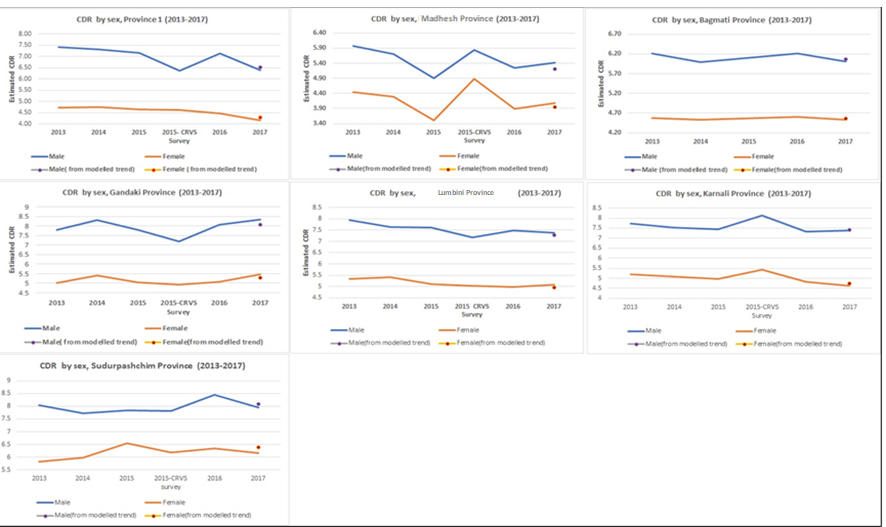
**

**Fig A3: Under-five mortality (*_5_q_0_*) by provinces and sex, Nepal, 2017**


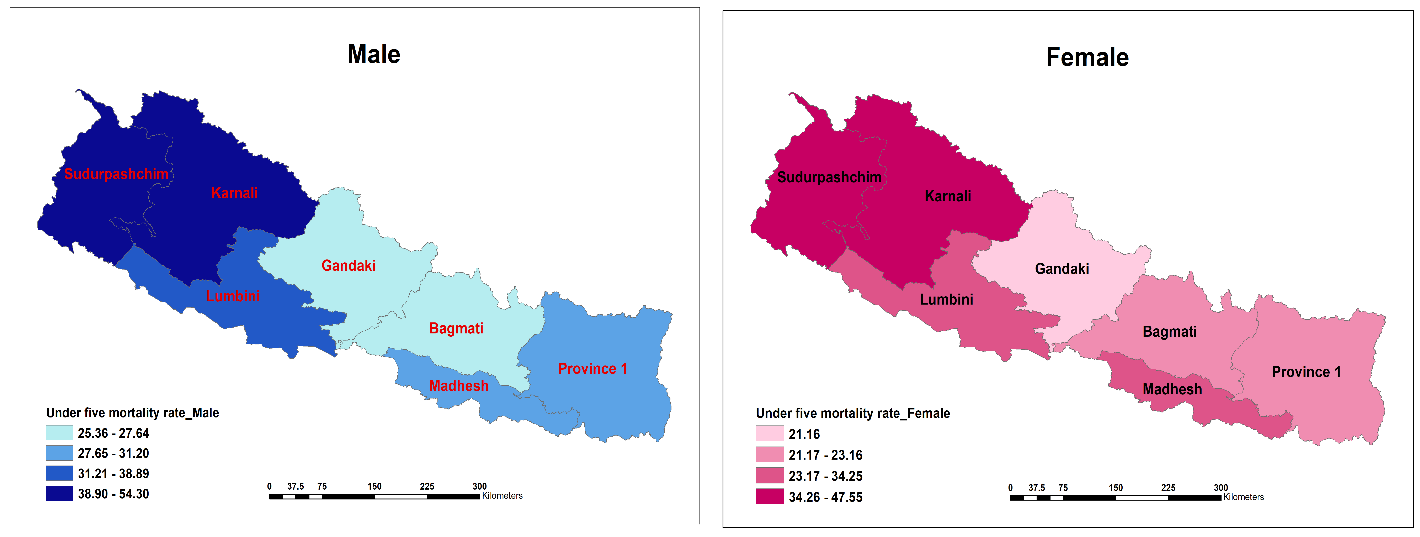


**Figure A4: Adult Mortality (_45_q_15_) by Provinces and Sex, Nepal, 2017**

**
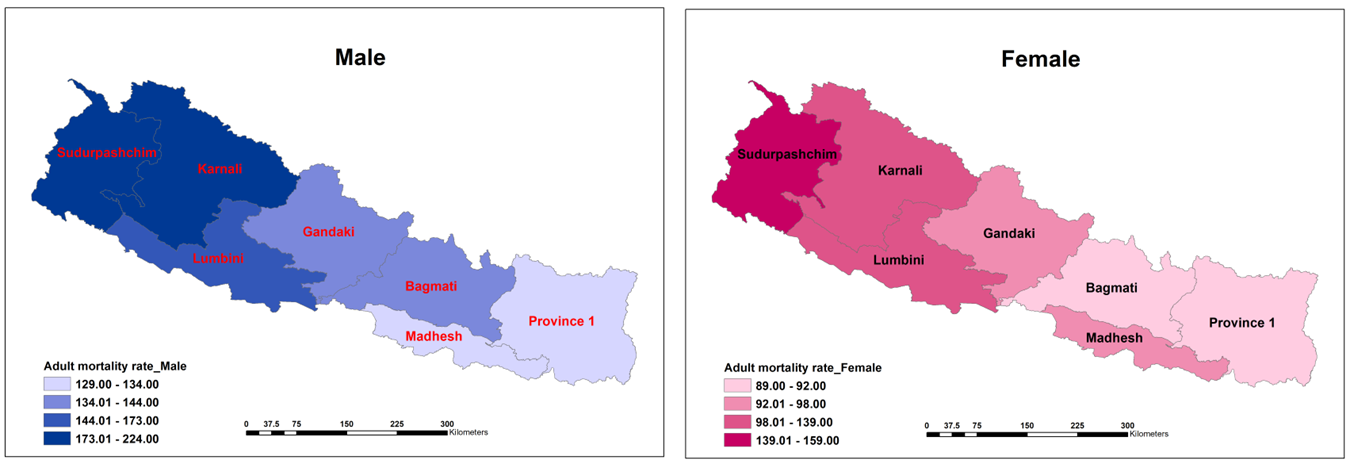
**

**Figure A5: Under-five mortality (per 1000) and poverty headcount rate (%) by provinces and sex, Nepal, 2017**

**
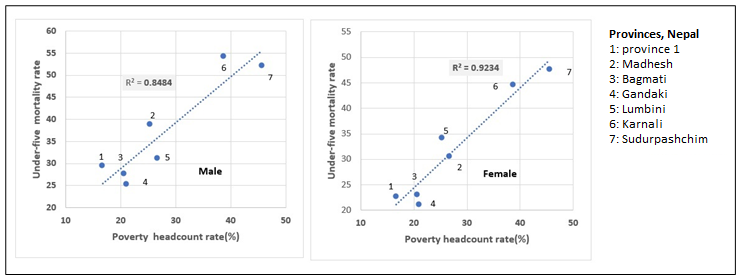
**

**Figure A6: Adult mortality (per 1000) and poverty headcount rate (%) by provinces and sex, Nepal, 2017**

**
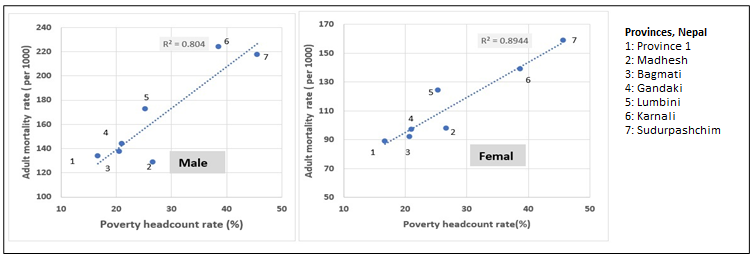
**

**Figure A7: Under-five mortality (per 1000) and poverty headcount rate (%) by ecological belts and sex, Nepal, 2017**


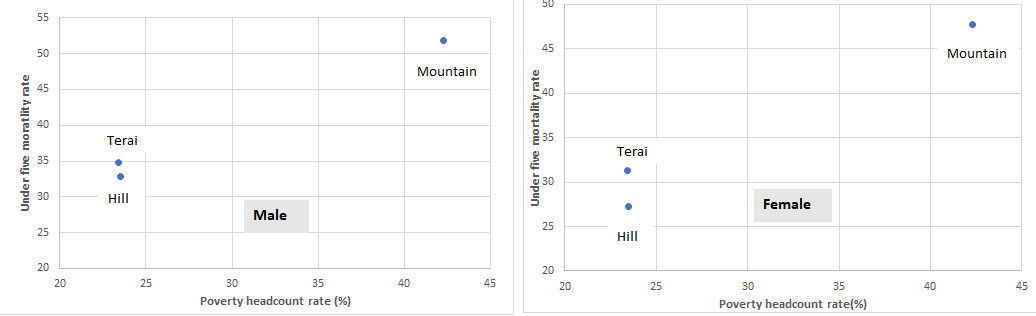


**Figure A8: Adult mortality (per 1000) and poverty headcount rate (%) by ecological belts and sex, Nepal, 2017**


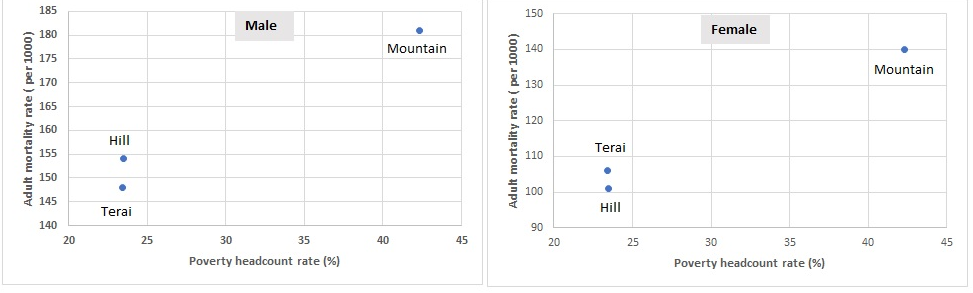


**References**

1. Pandey, S., Adair, T. 2022. Assessment of the national and subnational completeness of death registration in Nepal, *BMC Public Health*, 22: 429.
